# Determining the Temporal Profile of Intracranial Pressure Changes Following Transient Stroke in an Ovine Model

**DOI:** 10.3389/fnins.2019.00587

**Published:** 2019-07-09

**Authors:** Annabel J. Sorby-Adams, Anna V. Leonard, Levi E. Elms, Oana C. Marian, Jan W. Hoving, Nawaf Yassi, Robert Vink, Emma Thornton, Renée J. Turner

**Affiliations:** ^1^Adelaide Medical School, Adelaide Centre for Neuroscience Research, The University of Adelaide, Adelaide, SA, Australia; ^2^Department of Medicine and Neurology, Melbourne Brain Centre at the Royal Melbourne Hospital, University of Melbourne, Melbourne, VIC, Australia; ^3^Department of Radiology, Amsterdam UMC, University of Amsterdam, Amsterdam, Netherlands; ^4^Florey Institute of Neuroscience and Mental Health, Melbourne, VIC, Australia; ^5^Division of Health Sciences, University of South Australia, Adelaide, SA, Australia

**Keywords:** intracranial pressure, cerebral edema, stroke, large animal model, blood-brain barrier

## Abstract

**Background and Purpose:**

Cerebral edema and elevated intracranial pressure (ICP) are the leading cause of death in the first week following stroke. Despite this, current treatments are limited and fail to address the underlying mechanisms of swelling, highlighting the need for targeted treatments. When screening promising novel agents, it is essential to use clinically relevant large animal models to increase the likelihood of successful clinical translation. As such, we sought to develop a survival model of transient middle cerebral artery occlusion (tMCAO) in the sheep and subsequently characterize the temporal profile of cerebral edema and elevated ICP following stroke in this novel, clinically relevant model.

**Methods:**

Merino-sheep (27M;31F) were anesthetized and subject to 2 h tMCAO with reperfusion or sham surgery. Following surgery, animals were allowed to recover and returned to their home pens. At preselected times points ranging from 1 to 7 days post-stroke, animals were re-anesthetized, ICP measured for 4 h, followed by imaging with MRI to determine cerebral edema, midline shift and infarct volume (FLAIR, T2 and DWI). Animals were subsequently euthanized and their brain removed for immunohistochemical analysis. Serum and cerebrospinal fluid samples were also collected and analyzed for substance P (SP) using ELISA.

**Results:**

Intracranial pressure and MRI scans were normal in sham animals. Following stroke, ICP rose gradually over time and by 5 days was significantly (*p* < 0.0001) elevated above sham levels. Profound cerebral edema was observed as early as 2 days post-stroke and continued to evolve out to 6 days, resulting in significant midline shift which was most prominent at 5 days post-stroke (*p* < 0.01), in keeping with increasing ICP. Serum SP levels were significantly elevated (*p* < 0.01) by 7 days post-tMCAO.

**Conclusion:**

We have successfully developed a survival model of ovine tMCAO and characterized the temporal profile of ICP. Peak ICP elevation, cerebral edema and midline shift occurred at days 5–6 following stroke, accompanied by an elevation in serum SP. Our findings suggest that novel therapeutic agents screened in this model targeting cerebral edema and elevated ICP would most likely be effective when administered prior to 5 days, or as early as possible following stroke onset.

## Introduction

Worldwide, over 15 million people suffer a stroke each year, of which one third will die and one third will remain permanently disabled as a result (Feigin et al., 2017). Cerebral ischemia initiates a cascade of interrelated events precipitating brain tissue injury and cell death (Ayata and Ropper, 2002). Of these secondary injury processes, increased microvascular permeability and loss of structural integrity of the blood-brain barrier (BBB) are key to the development of cerebral edema, a pathological state in which fluid abnormally accumulates in the extracellular space of the cerebral parenchyma, resulting in an overall increase in brain volume. This leads to a subsequent rise in intracranial pressure (ICP), which has devastating consequences including infarct expansion, displacement of brain tissue, tonsillar herniation, and death (Steiner et al., 2001; Raslan and Bhardwaj, 2007; Battey et al., 2014; Kim et al., 2015).

Stroke patients often exhibit a progressive and slow evolution of brain injury, with cerebral edema and elevated ICP presenting some days following initial insult to tissue, most commonly around 3–5 days following onset (Hewitt and Ellory, 2012). Despite the devastating impact of cerebral edema and elevated ICP, however, existing treatments fail to address the underlying pathophysiology, thereby limiting their efficacy (Battey et al., 2014). Novel therapies that target the underlying cause of swelling and prevent the evolution of cerebral edema and elevated ICP are urgently required to improve post-stroke outcomes. To this end, our laboratory has identified that the tachykinin neuropeptide substance P (SP) is a key mediator in BBB dysfunction and development of cerebral edema following stroke (Turner et al., 2006, 2011; Turner and Vink, 2012) and traumatic brain injury (TBI) (Vink et al., 2003; Nimmo et al., 2004; Donkin et al., 2009; Corrigan et al., 2012). Specifically, SP release leads to neurogenic inflammation, manifesting following acute central nervous system (CNS) injury as increased BBB permeability and the development of cerebral edema. Furthermore, we have established that blockade of the receptor for SP, the tachykinin NK1 receptor (NK1-r), reduces both BBB permeability and cerebral edema in rodent models of stroke (Turner et al., 2011; Turner and Vink, 2012, 2014) and TBI (Donkin et al., 2009; Corrigan et al., 2012, 2016), suggesting that this is a common feature of acute brain injury.

An enhanced understanding of the underlying pathophysiology of cerebral edema and elevated ICP following stroke is essential in guiding the development of more effective therapies, and pre-clinical stroke studies are an essential step in this process. However, the poor clinical translation of stroke therapies from the laboratory to the clinic to date has emphasized the importance of appropriate species and model selection (Casals et al., 2011). The use of large intermediate species for these studies is paramount in the pre-clinical phase to improve translation to the clinic due to similarities in neuroanatomical structure, such as a large gyrencephalic brain that is closer in similarity to that of the human compared with many widely adopted small animal models. In light of this, a number of large animal species are used as pre-clinical stroke models, including sheep (Boltze et al., 2008; Wells et al., 2012, 2015), dogs (Qureshi et al., 2004; Cameron et al., 2008; Boulos et al., 2011; Christoforidis et al., 2011), pigs (Sakoh et al., 2000; Watanabe et al., 2007; Platt et al., 2014), and non-human primates (Nehls et al., 1986; Tagaya et al., 1997; Yuji et al., 2001; D’Ambrosio et al., 2004; Furuichi et al., 2007), each with their own inherent advantages and disadvantages (Sorby-Adams et al., 2018). Although gyrencephalic non-human primates are of great value in modeling the human condition, the cost, housing requirements and ethical considerations may preclude their use in large scale studies. Pigs are a widely used experimental species in models of cardiovascular (Suzuki et al., 2011; Kamoi et al., 2014) and TBI studies worldwide (Lighthall, 1988; Pfenninger et al., 1989; Ross et al., 1994; Duhaime et al., 2000; Manley et al., 2006), however, the Yucatan mini-pig is not available in Australia, and the rapidly increasing bodyweight of other strains can make long-term studies difficult. Sheep are an accessible experimental species and have been used extensively to model a number of neurological conditions, including non-accidental head injury (Sandoz et al., 2012; Anderson et al., 2014), TBI (Vink et al., 2008; Vink et al., 2017), and Huntington’s Disease (Reid et al., 2013; Morton et al., 2014). Although sheep have earned a reputation as unintelligent animals, the evidence is quite to the contrary, as sheep demonstrate excellent facial recognition, executive decision making and emotional processing, often comparable to humans on equivalent tasks (Tate et al., 2006; Keith et al., 2007; Morton and Avanzo, 2011). Specifically, the high proportion of white to gray matter in the sheep brain is of particular benefit when studying the pathophysiology of edema and ICP, given that edematous fluid accumulates in the white matter following ischaemic stroke (Jha, 2003; Mahajan and Bhagat, 2016). The strong fibrous tentorium cerebelli of the sheep, comparable with that of the human, also prevents the brain from moving downward to accommodate increased volume of the edematous brain, resulting in persistent elevations in ICP, midline shift and tonsillar herniation all of which are seen clinically. Additional advantages of large animal models, is the ability to take multiple serum and cerebrospinal fluid (CSF) samples from an individual animal over time which is not possible in rodents, as well as using clinical physiological monitoring equipment and magnetic resonance imaging (MRI). As such, large animal stroke models, including the sheep, are an excellent additional screening tool following evaluation in rodents prior to progression to clinical trials.

There are two models of ovine middle cerebral artery occlusion (MCAO) currently in use (Boltze et al., 2008; Wells et al., 2012) both of which involve permanent occlusion of the MCA. In the Boltze model (Boltze et al., 2008) the craniotomy is left open so that animals are effectively given decompression at the time of stroke, allowing for the investigation of long-term time-points without premature mortality, although intracranial pressure dynamics cannot be investigated. Conversely, in the Wells model (Wells et al., 2012, 2015) the craniotomy is closed following MCAO so that the evolution of ICP can be accurately studied. However, survival time-points beyond 24 h are not possible in this model due to the resulting malignant MCA infarction and ethical requirement to maintain animals under anesthesia for the duration of the experiment. As such, there is a clear requirement for an ovine MCAO stroke model where the MCA is temporarily occluded to study the effects of reperfusion injury and longer time-points following stroke onset. Furthermore, there is an increasing incidence of patients who successfully recanalize following stroke onset. This is largely due to the increased use of endovascular clot retrieval for reperfusion of large strokes, such as occlusion of the internal carotid artery (ICA) or M1 segment of the MCA (Goyal et al., 2016). The mechanical recanalization of the MCA able to be produced in a transient MCAO model closely aligns with the vascular reperfusion achieved with endovascular thrombectomy, and thus may be more representative of the human condition.

Accordingly, the aims of the present study were to: (1) develop a survival model of ovine MCAO stroke with reperfusion; (2) characterize the temporal profile of intracranial pressure changes following stroke; and (3) determine the contribution of SP to BBB permeability and cerebral edema that develops in this model. By determining the clinical course of ICP in this model, an optimal time window for administration of novel therapeutic agents targeting elevated ICP due to cerebral edema can be established.

## Materials and Methods

### Experimental Design

A total of 58 (*n* = 27M;31F) merino sheep (weight range 65 ± 7 kg; age 18–36 months) were used in this study. Animals (3M and 3F/time point) were pre-operatively randomized to daily post-stroke survival time points ranging from 1–7 days. Animals allocated to the post-stroke time-points underwent two surgeries: tMCAO surgery with recovery, and ICP monitoring on the designated day post-stroke. An additional group of animals underwent the surgical procedure without MCAO and acted as sham controls (*n* = 4F). These animals had ICP continuously recorded for 4 h immediately following the sham surgical procedure. Finally, an additional follow up cohort of 12 animals (*n* = 6F;6M) underwent stroke surgery and were included to obtain serum and CSF samples over the 7-day post-stroke time-course. This was not possible in the main stroke cohort due to the risk that repeat CSF sampling might potentiate changes in intracranial dynamics leading to inaccurate measurements of ICP. All animals were group housed in conventional sheep paddocks for a minimum of 2 weeks prior to surgery to allow for acclimatization. Animals were then moved to individual indoor pens 48 h prior to surgery and fasted over night for a minimum of 12 h prior to the procedure.

### Experimental Procedure

#### Animal Preparation and Anesthesia

Anesthesia was induced with a combination of intravenous ketamine (0.05 ml/kg, 100 mg/kg Injection, CEVA, Australia) and diazepam (0.08 ml/kg, 5 mg/ml Injection, Pamlin, CEVA, Australia). Following induction, animals were intubated via endotracheal tube insertion to ensure airway patency for ventilation. Animals were then positioned on the operating table on their left side, with hind limbs exposed for arterial access. An arterial catheter was inserted in the left hind leg for blood gas sampling and a cannula inserted into the external jugular vein for fluid and drug administration. Intraoperative anesthesia was maintained via a combination of inhalational isoflurane (Henry Shein, Australia; 1.5–2.0%) in 100% oxygen and intravenous (jugular) ketamine at an infusion rate of 4 mg/kg/h. Crystalloid fluids were administered throughout surgery via continuous intravenous infusion with compound sodium lactate (Baxter Health, Australia) to assist hydration and ionic balance. In addition, intraarterial sodium chloride (Baxter Health, Australia) was infused at a rate sufficient to ensure arterial catheter patency. Following surgery, animals were recovered and treated with subcutaneous antibiotic Rilexine (1 ml/10 kg every 12 h, 15 g/100 ml Cephalexin, VIBRAC, Australia), NSAID Carprieve (0.7 mg/kg, 50 mg/ml every 12 h, Carprofen, Norbrook, Australia) and opioid Temgesic (1.0 ml, 324 ug/ml Buprenorphine hydrochloride, one dose post-operatively, Reckin Benckiser, Australia) for pain relief. Antibiotic and NSAID treatment was continued for a minimum of 3 days following surgery, with additional opioid and/or NSAID administered as required thereafter for pain management.

#### Surgical Approach

**FIGURE 1:**
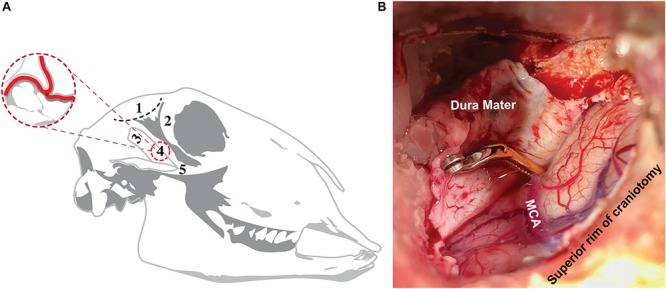
Surgical approach to ovine transient MCAO. Surgical approach **(A)**, showing site of incision (1; black dashed line) posterior to the orbital rim (2) and superior to the coronoid process (3). This approach enabled the skull base to be accessed without disrupting the coronoid process or zygomatic arch (5), allowing for craniotomy (4) and direct access to the MCA, whilst ensuring post-operative recovery of the animal and the ability to masticate following the procedure. Superior view of craniotomy site **(B)** showing the aneurysm clip applied to the proximal MCA.

A model of ovine stroke was modified from previously published works (Wells et al., 2012, 2015) in order to achieve tMCAO with reperfusion (Figure 1). This modification enabled the recovery of animals from surgery and the study of longer time-points (beyond 24 h) following stroke. In this particular approach, it was necessary to keep the coronoid process of the mandible intact and preserve the surrounding temporalis muscle to ensure animals were able to masticate following surgery. To achieve this, a 50 mm curved horizontal incision was made posterior to the orbital rim of the right eye and terminating anterior to the ear. Upon incision, the temporalis muscle was retracted and the coronoid process elevated to expose the underlying skull. Cervical retractors (Codman) were inserted and blades adjusted to provide space for a small craniotomy to be drilled at the junction of the parietal and squamous temporal bones with a high-speed pneumatic drill using a 5 mm cutting burr (Midas Rex, Medtronic, MN, United States). Following craniotomy, a horse shoe durotomy was performed, the brain exposed and the anterior temporal lobe gently retracted to expose the right proximal MCA. A small amount of CSF was aspirated to ensure sufficient visualization of the proximal MCA, however, this was kept to a minimum to reduce the impact on intracranial volume. Gentle dissection of the MCA from the arachnoid mater was performed to allow for a temporary aneurysm clip (Aesculap) to be applied proximal to the MCA bifurcation using a clip applicator (YASARGIL Aneurysm clip system, Aesculap). Successful clipping of the MCA resulted in an immediate decrease in vessel caliber, confirming reduced blood flow. The dura was then gently re-opposed onto the brain and the craniotomy site filled with sterile saline to prevent dehydration of the underlying cortical tissue and the surgical site draped. The clip was left *in situ* for 2 h and subsequently removed with clip applicators to achieve reperfusion. Visual confirmation of reperfusion was noted when the caliber of the vessel increased and blood returned to the distal MCA. Successful reperfusion also was confirmed on magnetic resonance angiography (MRA) MRI performed at the pre-selected timepoints.

Following reperfusion, the dura was approximated and interleaved with synthetic dura (Durepair, Medtronic, Australia) to cover the exposed brain. Dural closure was then achieved with ethyl cyanoacrylate (Bostik, Australia), and the bone previously removed reinserted into the craniotomy site and reinforced with dental acrylic cement (Sledgehammer, Keystone, Germany) to create a watertight seal, thus restoring intracranial dynamics. The surgical site was then treated with 1.0 ml 0.5% local anesthetic Marcain (bupivacaine hydrochloride 5 mg/ml, AstraZeneca, Australia) and closed using a 0.1 Vicryl braided polyglactin Suture (ETHICON, Australia) using the horizontal mattress suture technique of wound closure to minimize risk of infection. The animal was removed from anesthesia, extubated and pain relief administered prior to transfer into a post-operative recovery pen. Mean arterial blood pressure (MABP) was monitored at 5-min intervals throughout surgery via application of a pediatric blood pressure cuff (Delfi Medical, Canada) on the upper left front limb. Blood gas samples (Machine AVL Scientific Corporation United States, Manufactured by Hersteller, OPTI Critical Care Analyzer–Model OPTI3, Serial No. OP3-2759) were acquired hourly via arterial catheter sampling to determine pO_2_, pCO_2_, hematocrit, hemoglobin, pH, Na^+^ and K^+^ levels. End tidal CO_2_ was also recorded at regular intervals. Animals were maintained on a heating pad throughout all surgical procedures and rectal temperature checked hourly to ensure maintenance of body temperature within normal ovine limits (37.5–39.0°C).

#### ICP Measurement

At 4 h prior to the pre-determined post-stroke time-point, or immediately following surgery in shams, anesthetized (using aforesaid isoflurane and ketamine maintenance protocols) animals were placed prone in the sphinx position on the operating table. Bilateral burr holes were drilled into the left and right parietal bones, posterior to the coronal suture and approximately 20 mm from the sagittal suture. The dura was perforated and skull bolts secured, following which Codman microsensor ICP probes (Codman Neuro, DePuy Synthes, Australia) were inserted to an approximate depth of 15 mm into the brain parenchyma and ICP recorded continuously for a period of 4 h post-MCAO induction or sham surgery (LabChart Reader, ADInstruments, v. 8.1.1). Two-point calibration (0 and 100 mmHg) was performed prior to insertion of probes and at the end of the recording period to confirm measurement accuracy. ICP readings selected for final analysis (left or right) were based on calibration data, with readings preferentially selected from the right hemisphere probe. Raw ICP data underwent a logarithmic exponential transformation as previously described and was expressed as geometric mean ± SEM (Matthews et al., 1990; Wells et al., 2015).

#### MRI

Following the 4-h ICP monitoring period, animals were moved to a 1.5T Siemens Syngo2004A (Siemens AG, Munich, Germany) MRI scanner, during which they were maintained under general anesthesia (3% isoflurane). Sequences were acquired including time-of-flight MRA (TOF MRA; TR 26 ms, TE 3.69 ms, slice thickness 0.50 mm), diffusion weighted imaging (DWI; TR 5600 ms, TE 80 ms, slice thickness 3.0 mm), fluid attenuated inversion recovery (FLAIR; TR 5000 ms, TE 386 ms, slice thickness 0.9 mm), T1-weighted imaging (TR 2300 ms, TE 2.58ms, slice thickness 0.9 mm) and T2-weighted imaging (TR 3200 ms, TE 410 ms, slice thickness 0.9 mm). T1-weighted post-contrast images were also acquired following intravenous administration of 1 mL/kg gadolinium-diethylene-triamine-pentaacetic acid (GAD) (Magnevist, Bayer HealthCare, Germany) to determine the extent of BBB breakdown on MRI.

The degree of midline shift was used as a surrogate marker for the anatomical sequalae of cerebral edema. Shift from the midline was assessed using axial T2-weighted scans and measured in mm from the septum pellucidum at the level of the foramen of Monro (Horos DICOM image viewer v3.1.1). To calculate cerebral edema, coronal FLAIR images were analyzed using Horos DICOM image viewer (v3.1.1). After optimal adjustment of brightness and contrast, edema volume was determined from sequences using computer-aided manual tracing of the hyperintense lesion by a blinded assessor. The areas were then summed and multiplied by the slice thickness to give a total volume in cm^3^.

To calculate infarct volume, segmentation tools in ITK-SNAP (v3.7) were used to perform semi-automated segmentation of the MRI diffusion lesions using diffusion-weighted images (Yushkevich et al., 2006). A combination of “three-dimensional active contour segmentation” and subsequent manual post-processing of the segmentation while adjusting image thresholds was performed to maximize reproducibility whilst excluding artifacts. The “three-dimensional active contour segmentation” consisted of multiple steps: First, in the pre-segmentation phase, independent component analysis automatically segmented parts of the DWI image and these were manually identified as foreground or background. To obtain an optimal distinction between foreground and background, thresholds of the image windows were adjusted. After thresholding, a “speed image” with a separate foreground and background was created. Next, in the active contour phase, seed regions were manually placed within the region of interest (ROI). These seeds were then automatically grown within the ROIs to form the temporary segmentation. Subsequently, areas that were not automatically included in the “active contour segmentation,” were manually included in the follow-up infarct segmentation. The total volume of the segmentation was exported and reported in cm^3^ for analysis.

#### Perfusion and Histological Examination

Following MRI, intravenous heparin (5000 I.U./5 ml; Pfizer, NY, United States) was administered and animals humanely euthanized via common carotid perfusion fixation with cold TRIS-buffered saline under isoflurane anesthesia. The brains were subsequently removed and sliced into 10 mm coronal slices using a custom-made sheep brain matrix. Sections were then immersion fixed in 10% neutral-buffered formalin for a minimum of 14 days prior to being processed, embedded in paraffin wax and sectioned coronally at 5-micron intervals to identify albumin, SP, and caveolin-1 via immunohistochemistry (Table 1).

**TABLE 1:** Immunohistochemistry protocols.

**Antibody**	**Dilution**	**Retrieval**	**Brand**	**Catalog no.**
Albumin	1:2000	No retrieval	Dako	A0001
SP	1:5000	Citrate	Abcam	ab14184
Caveolin-1	1:500	EDTA	Cell Signaling Technologies	3238

#### Serum and CSF Collection

Blood samples were collected (BD-Vacutainer^®^-SST^TM^-II-Advance-Tube) from animals (*n* = 12, 6M;6F) pre-injury and at 4, 8, 12, and 16 h post-stroke and on days 1, 3, 5, and 7 via jugular venipuncture. Following collection, samples were centrifuged (10 min at 2000*g*) and serum extracted into 10 × 500 μl aliquots and stored at −80°C. CSF was collected via lumbosacral tap prior to induction of stroke and on days 1, 3, and 6 thereafter. Note that this was performed in the follow up cohort, and these animals did not undergo ICP monitoring so there were no concerns regarding multiple CSF draws and the associated effects on ICP measurement accuracy. Prior to collection, animals were induced with combination diazepam and ketamine as previously described. The animals were then placed in the prone position on a surgical table and the sagittal plane of the animals vertebrae manipulated so that it was perpendicular with the horizontal plane of the bench. The animal was shaved to allow for clear access to the lumbosacral joint and appropriate visualization for landmarks. Palpation of the hips was performed to determine the correct sagittal plane orientation of the spine and the optimal site for CSF collection was determined via identification of the midline depression between the last lumbar and first sacral vertebrae. The area was cleaned using a 4% chlorhexidine preoperative surgical scrub brush (BD E-Z Scrub^TM^), povidone-iodine applied and site surgically prepared. A steriseal spinal needle with stylet (Becton Dickinson Medical Technology) was inserted perpendicular to the skin and the needle advanced slowly through the subcutaneous tissue and interarcuate ligament until a sudden drop in resistance was felt indicating the advancement through the dural membrane and entry into the subarachnoid space. The stylet was then removed and 2 ml of CSF aspirated via a syringe and deposited into a 3 ml container; sample placed immediately on ice for 20 min, centrifuged (15 min at 3000*g*), extracted into 3 × 500 μl aliquots and stored at −80°C.

#### Analysis of Serum and CSF

Serum and CSF samples were used to quantify expression of SP using enzyme-linked immunosorbent assay (ELISA) according to the manufacturer’s instructions (Abcam; KIT-ab133029). All samples were diluted 1:4 in assay buffer for appropriate compliance with each individual plate standard curve. Standards, serum and CSF test samples were run and the plate immediately analyzed following addition of the stop solution using a spectrophotometric microplate reader (Synergy HTX, multi-mode reader). At optical density of 405 nm light absorbance was calculated to give an inverse reading of SP proportional to its captured measure on the plate.

### Statistical Analysis

All data are expressed as mean ± SEM. Physiological data (MABP, pH, pO_2_, and pCO_2_) were analyzed using one-way analysis of variance (ANOVA) followed by Tukey’s *post hoc* tests (Prism v.8.0.1, Graphpad, CA, United States). ICP, infarct volume, cerebral edema and midline shift data were analyzed by one-way ANOVA with multiple comparisons followed by Tukey’s *post hoc* tests. ELISA evaluation was performed for each plate using individually generated standard curves and an *R*-value of 0.9709 was accepted to improve the efficacy of inter-plate accuracy. ELISA values were then analyzed via one-way ANOVA with Tukey’s *post hoc*. Pearson’s correlations were run between midline shift and cerebral edema, cerebral edema and ICP and midline shift and ICP to determine the relationship between variables. A two-way ANOVA with Sidak’s multiple comparison test was run to determine gender differences in physiological variables during both surgery and ICP monitoring. Gender discrepancies were also established for infarct volume, cerebral oedema, midline shift and ICP in addition to CSF and serum SP via two-way ANOVA with Sidak’s *post hoc*. A *p*-value < 0.05 and *R*-value > 0.04 was considered significant.

## Results

### Surgery, Mortality, and Post-operative Course

**FIGURE 2:**
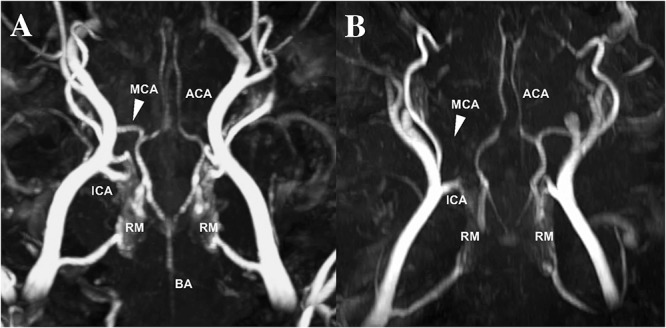
MCA reperfusion following transient MCAO **(A)** compared to permanent MCAO **(B)** on time-of-flight MRA. Evidence of vessel patency, indicated by the white arrow, following clip removal in transient MCAO animals compared to permanent MCAO animals, where the vessel cannot be visualized MRA (data not reported). MCA, middle cerebral artery; ACA, anterior cerebral artery; ICA, internal carotid artery; BA, basilar artery; RM, rete mirabile.

There was no premature mortality of animals in the present study. One animal was excluded due to insufficient reperfusion following MCAO as seen on MRA, resulting in a permanent rather than a transient occlusion (Figure 2). In all other temporary occlusions of the MCA reperfusion was successfuly achieved and confirmed on MRA. A further four animals were excluded due to significant anesthetic difficulties which confounded the experimental course (*n* = 2 in 2 days group; *n* = 2 in 3 days group). Specifically, the excluded animals were unstable throughout the induction of stroke surgery, necessitating repeat boluses of ketamine and diazepam and an increased rate of isoflurane to maintain surgical levels of anesthesia, which resulted in significantly lowered MABP, decreased cerebral perfusion pressure and ultimately profound infarct expansion.

All animals were able to use their jaw and masticate to eat following surgery. A comprehensive behavioral analysis was beyond the scope of this study, however, all animals in the ICP cohort were observed for jaw alignment and eating behavior, the presence/absence of fetlock weakness and demeanor throughout the post-stroke period. A total of 9/42 animals demonstrated evidence of jaw misalignment following stroke surgery, which persisted to the end of the experiment in 3/9 animals. Although 25/42 animals showed reduced food intake post-stroke when compared to their pre-stroke daily intake, body weight remained within acceptable limits (< 15% reduction from pre-stroke weight). A total of 14/42 animals showed fetlock weakness following stroke, manifesting as knuckling of the distal forelimb which was most commonly observed on day 1 and typically resolved by day 4 post-stroke. A decline in demeanor, as demonstrated by a lowered head position and droopy ears, was observed in 26/42 animals and was most prevalent at days 5–7 post-stroke, coinciding with elevated ICP.

### Physiological Parameters

Physiological variables for pCO_2_, MABP, and pH were within normal limits for all groups throughout the duration of the experiment (Table 2). As animals were maintained on 100% oxygen (oxygen 6.0 L/min) through the course of surgery and ICP monitoring this resulted in pO_2_ values that were significantly above the normal clinical range of 90–100 mmHg. These observations, however, were observed across all experimental groups. No significant difference (*p* > 0.05; Table 1) in pO_2_, pCO_2_, or pH were observed amongst groups during stroke surgery or the 4-h period of ICP monitoring. There was however a statistically significant decrease in MABP during the ICP monitoring period seen at 1-day post-stroke compared with 4 (*p* = 0.04) and 7 (*p* = 0.01) days.

**TABLE 2:** Surgical and intracranial pressure monitoring physiological parameters.

	**Surgery day**			**ICP monitoring**			**Surgery day**			**ICP Monitoring**		
				
**Group**	**Male**	**Female**	**All animals**	**Male**	**Female**	**All animals**	**Male**	**Female**	**All animals**	**Male**	**Female**	**All animals**
		
	**PCO_2_ (mmHg) ± SEM**						**PO_2_ (mmHg) ± SEM**					
Sham		43.19 ± 3.43						112.77 ± 5.75				
1 Day	41.19 ± 3.39	39.83 ± 2.07	40.51 ± 1.80	36.08 ± 1.61	37.42 ± 2.32	36.75 ± 1.30	213.94 ± 50.81	213.08 ± 66.77	213.51 ± 37.52	294.36 ± 64.89	191.64 ± 38.27	243 ± 40.78
2 Day	43.83 ± 2.05	45.25 ± 2.84	44.54 ± 1.60	38.33 ± 1.02	41.42 ± 2.14	39.88 ± 1.27	200.17 ± 37.13	83.67 ± 10.83	141.92 ± 31.27	166.67 ± 14.21	250.92 ± 62.62	208.79 ± 34.34
3 Day	46.58 ± 1.72	46.25 ± 1.50	46.42 ± 1.02	47.28 ± 2.98	40.25 ± 1.75	44.47 ± 2.43	289.50 ± 96.27	187.0 ± 38.56	238.25 ± 51.73	241.81 ± 126.13	237.38 ± 72.13	240.03 ± 72.76
4 Day	46.81 ± 3.13	43.0 ± 3.47	44.90 ± 2.26	42.89 ± 0.45	38.17 ± 4.19	40.53 ± 2.16	193.00 ± 73.93	113.50 ± 8.75	153.25 ± 37.74	304.67 ± 47.92	163.0 ± 17.10	233.83 ± 39.00
5 Day	50.08 ± 3.17	44.33 ± 2.51	47.21 ± 2.22	41.58 ± 0.79	39.58 ± 2.19	40.58 ± 1.13	193.83 ± 30.12	188.47 ± 57.77	191.15 ± 29.16	228.17 ± 70.65	251.33 ± 29.37	239.75 ± 34.61
6 Day	45.42 ± 2.38	41.33 ± 1.62	43.38 ± 1.58	47.75 ± 1.75	42.83 ± 1.96	45.29 ± 1.61	342.33 ± 101.85	217.58 ± 76.33	279.96 ± 63.39	319.56 ± 122.91	422.67 ± 57.68	371.11 ± 64.95
7 Day	47.67 ± 1.88	44.08 ± 2.24	45.80 ± 3.76	42.75 ± 2.89	46.25 ± 0.88	44.50 ± 1.56	172.08 ± 79.94	116.50 ± 8.62	144.29 ± 38.04	313.03 ± 83.77	280.83 ± 48.43	296.93 ± 43.87

	**MABP (mmHg) ± SEM**						**pH ± SEM**					

Sham		79.35 ± 6.27						7.46 ± 0.02				
1 Day	84.61 ± 12.54	86.17 ± 8.80	85.39 ± 6.86	97.25 ± 8.76	77.64 ± 8.54	87.44 ± 7.01	7.43 ± 0.03	7.44 ± 0.01	7.43 ± 0.01	7.42 ± 0.01	7.42 ± 0.03	7.42 ± 0.01
2 Day	74.17 ± 0.87	92.92 ± 4.01	106.98 ± 25.30	88.50 ± 11.35	107.39 ± 17.88	96.44 ± 10.66	7.43 ± 0.02	7.45 ± 0.04	7.44 ± 0.02	7.43 ± 0.03	7.48 ± 0.03	7.45 ± 0.02
3 Day	75.42 ± 5.47	91.42 ± 4.03	83.42 ± 4.69	93.75 ± 14.42	99.88 ± 12.63	96.20 ± 8.97	7.41 ± 0.04	7.41 ± 0.01	7.41 ± 0.02	7.39 ± 0.04	7.41 ± 0.03	7.40 ± 0.02
4 Day	92.75 ± 3.82	84.17 ± 3.99	88.46 ± 3.13	116.33 ± 10.19	127.50 ± 6.91	121.92 ± 6.05^*^	7.41 ± 0.02	7.44 ± 0.02	7.42 ± 0.02	7.35 ± 0.05	7.48 ± 0.02	7.41 ± 0.04
5 Day	96.75 ± 1.32	80.97 ± 13.45	88.86 ± 7.00	117.92 ± 7.58	104.75 ± 1.98	111.33 ± 4.58	7.38 ± 0.02	7.43 ± 0.07	7.40 ± 0.02	7.47 ± 0.02	7.43 ± 0.07	7.45 ± 0.03
6 Day	78.67 ± 9.04	91.17 ± 2.98	119.56 ± 6.87	122.67 ± 2.85	108.42 ± 5.21	115.54 ± 4.15	7.39 ± 0.03	7.45 ± 0.01	7.42 ± 0.02	7.40 ± 0.02	7.44 ± 0.03	7.42 ± 0.02
7 Day	114.17 ± 11.28	84.25 ± 10.31	99.21 ± 9.56	137.42 ± 17.12	116.75 ± 10.33	127.08 ± 10.07^*^	7.40 ± 0.02	7.42 ± 0.02	7.41 ± 0.01	7.48 ± 0.02	7.44 ± 0.03	7.46 ± 0.02

^*^p < 0.05 compared to day 1.

### Intracranial Pressure

**FIGURE 3:**
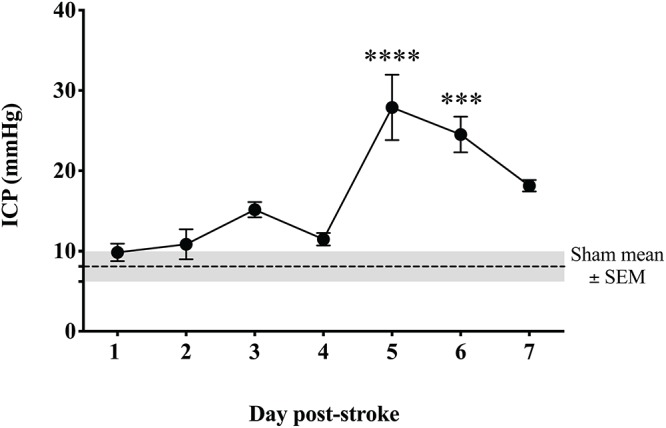
Temporal profile of ICP following transient MCAO. ICP was significantly elevated at 5 days (*p* < 0.001) and 6 days (*p* < 0.05) post-stroke when compared to sham. By 7 days, ICP began to resolve though remained elevated compared to sham (*p* < 0.05). ^∗∗∗∗^*p* < 0.0001; and ^∗∗∗^*p* < 0.001 compared to sham. *n* = 6/time-point, *n* = 4 sham.

Intracranial pressure remained within normal limits for the duration of the 4-h monitoring period in sham animals (8 ± 2 mmHg). From 1 to 4 days following tMCAO, ICP levels were comparable to sham (*p* > 0.05; Figure 3). However, by 5 days post-stroke, ICP was significantly elevated (28 ± 10 mmHg; *p* < 0.0001) from sham levels and post-stroke days 1–4 (*p* < 0.0001). ICP remained significantly elevated out to 6 days post-stroke when compared to shams (25 ± 5 mmHg; *p* = 0.0001) and 1 (*p* = 0.0001), 2 (*p* = 0.0003), 3 (*p* = 0.03) and 4 day (*p* = 0.0007) post-stroke time points. By 7 days, ICP began to decline (18 ± 2 mmHg; *p* = 0.02 when compared to 5 days), but remained significantly elevated compared to sham (*p* = 0.04), although not significantly different (*p* > 0.05) from recordings on days 1, 2, 3, 4, and 6 post-stroke.

### MRI

**FIGURE 4:**
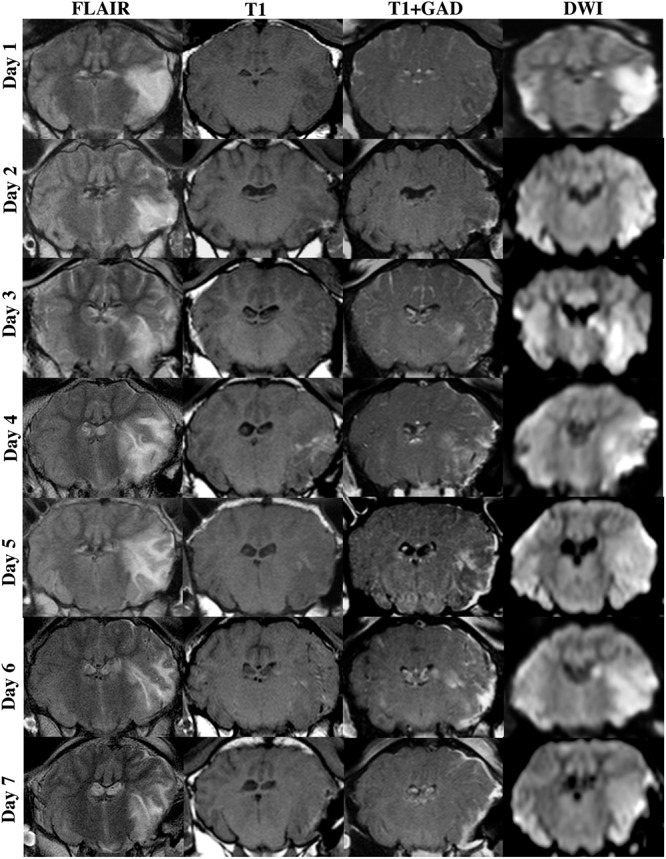
MRI findings following transient MCAO. Cerebral edema, as shown in coronal FLAIR MRI images, evolved from 1–6 days following stroke, beginning to resolve by 7 days. This evolution in cerebral edema was associated with enhanced extravasation of GAD across the BBB as seen on T1 weighted post-contrast series (T1+GAD) when compared to pre-contrast T1 series. Lesion volume, as shown in DWI MRI images, was comparable across time-points following stroke.

**FIGURE 5:**
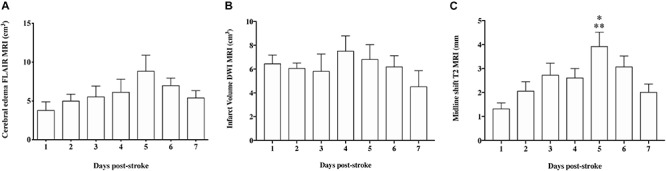
Quantification of infarct volume, cerebral edema, and midline shift on MRI. **(A)** There was no significant difference (*p* > 0.05) in infarct volume at any of the time-points following stroke. **(B)** The evolution of cerebral edema showed a similar pattern to ICP, however, changes were not significant (*p* > 0.05). **(C)** Midline shift was significantly greater at 5 days when compared to 1 (<0.01) and 7 (*p* < 0.05) days post-stroke. Data shown as mean ± SEM. ^*^p < 0.05 compared to 7 days, ^∗∗^*p* < 0.01 compared to 1 day. *n* = 6/time-point.

Magnetic resonance imaging was normal in sham animals, with no evidence of infarction, cerebral edema or midline shift (data not shown). At each time-point following stroke there was evidence of MCA recanalization following clip removal as seen on MRA, with the aforementioned exception of one animal that was excluded due to persistent MCAO (Figure 3). All stroke animals showed evidence of hyperintensity on T2-weighted and FLAIR images in the territory MCA, indicative of the infarct core and surrounding extracellular edema (Figure 4). There was no significant difference in infarct volume at any of the time points post-stroke as measured on DWI MRI (Figure 5A).

Cerebral edema evolved over time from 1 to 6 days post-tMCAO, with the largest volume of cerebral edema seen at 5 (8.86 ± 2.03 cm^3^) and 6 days (7.01 ± 0.9 cm^3^). This increase in the volume of edema was accompanied by increased extravasation of GAD across the barrier, as seen in T1 post-contrast sequences, indicative of BBB breakdown (Figure 4). However, although a qualitative evolution of cerebral edema post-stroke was apparent (Figure 4), accompanied by increased GAD contrast, there was no statistically significant difference between time-points (Figure 5B; *p* > 0.05). Midline shift was evident in all stroke animals, the degree of which evolved over time following stroke (Figure 5C) and was most prominent at 5 days post-stroke (3.92 ± 0.59 mm) when compared to 1 (*p* = 0.002) and 7 days (*p* = 0.04), in keeping with ICP findings. No animals showed evidence of tonsillar herniation or brain stem compression on MRI (data not shown).

**FIGURE 6:**
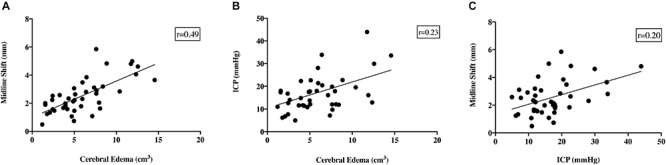
Relationship between ICP, cerebral edema, midline shift and infarct volume. There was a moderate positive correlation seen between cerebral edema and midline shift (*r* = 0.49, **A**). Despite this, there was no correlation seen between ICP and cerebral edema (*r* = 0.23, **B**), or ICP and midline shift (*r* = 0.20, **C**). NB data from all time points was used for comparison between variables.

Despite the fact that cerebral edema findings were not significantly different between time points, there was a moderate positive correlation seen between midline shift and cerebral edema calculations (*r* = 0.49; Figure 6A). However, there was no positive correlation seen between ICP and cerebral edema (*r* = 0.23; Figure 6B) or ICP and midline shift (*r* = 0.20; Figure 6C).

### SP ELISA

**FIGURE 7:**
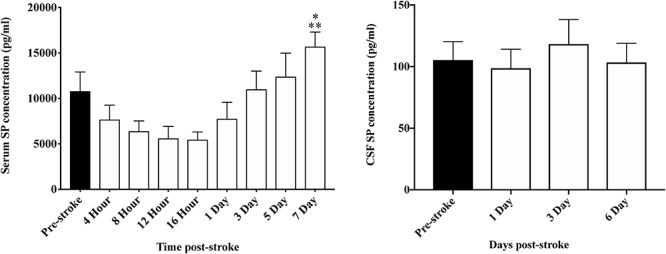
Temporal profile of serum and CSF SP levels following stroke. At 7 days post-stroke, SP in the serum was significantly elevated compared with levels seen at 4 h (*p* < 0.05), 8, 12, and 16 h (*p* < 0.01), and 1 day (*p* < 0.05) following stroke. Furthermore, despite a qualitative increase, SP concentration pre-stroke was comparable to day 7 post-stroke. In the CSF, there was no significant difference seen in SP levels at days 1, 3, and 6 post-stroke (*p* > 0.05). Data shown as mean ± SEM. ^*^p < 0.05 compared to 4 h and 1 day; ^∗∗^*p* < 0.01 compared to 8, 12, and 16 h. *n* = 12/time-point serum; *n* = 8/time-point CSF.

A reduction in serum SP was observed between 4 and 16 h following transient MCAO, however, changes were not significant (*p* > 0.05; Figure 7). At 7-days post-stroke, SP levels were significantly elevated compared to 16 h (*p* = 0.002) and days 1 (*p* = 0.04), 4 (*p* = 0.04), 8 (*p* = 0.008) and 12 (*p* = 0.003) following occlusion. Despite a qualitative increase, the concentration of serum SP at day 7 was not significantly elevated (*p* > 0.05) when compared to pre-stroke measurements. Furthermore, despite a relative increase in SP seen in the serum post-stroke, no significant changes (*p* > 0.05) in SP levels in the CSF (Figure 7) were observed at any time-point following MCAO.

### Gender Comparisons

**FIGURE 8:**
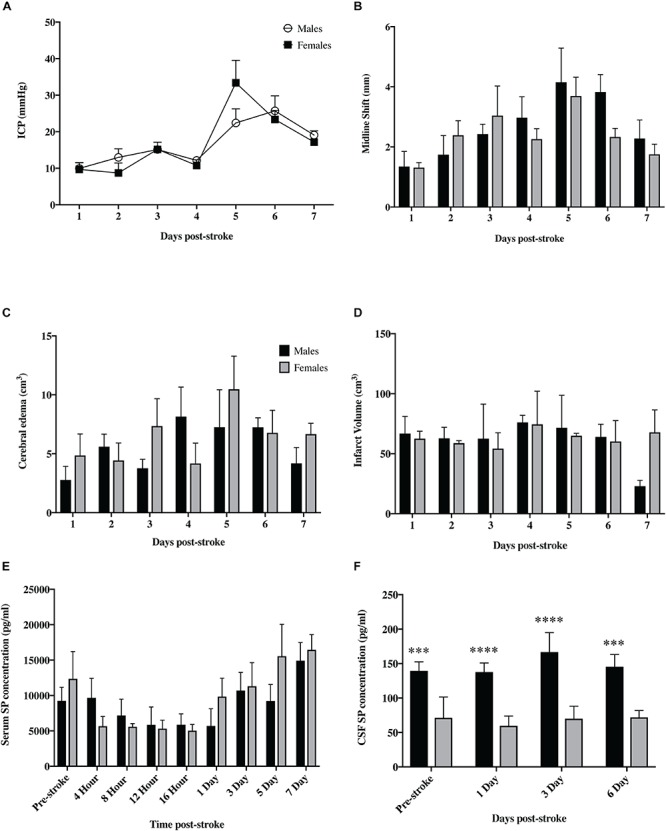
Sex differences following tMCAO. There was no significant difference in ICP **(A)** seen between sexes at any of the post-stroke time points (*p* > 0.05). Furthermore, there was no significant difference seen between males and females in infarct volume **(B)**, cerebral edema **(C)**, or midline shift **(D)** (*p* > 0.05). SP levels in the serum **(E)** were not significantly different between sexes (*p* > 0.05) but were significantly elevated in the CSF **(F)** of female animals compared to males at both pre and post-stroke time points (*p* < 0.01). Data shown as mean ± SEM. ^∗∗∗^*p* < 0.01 and ^****^p < 0.0001 females compared to males. *n* = 3/time-point ICP infarct volume, midline shift and edema; *n* = 6/time-point for serum; *n* = 4/time-point CSF.

No differences in pCO_2_, pO_2_, MABP or pH were seen between sexes during either stroke surgery or ICP monitoring (*p* > 0.05; Table 2). There was no significant difference in ICP between male and female animals at any of the post-stroke time-points (*p* = 0.93; Figure 8A). Furthermore, there was no significant difference in infarct volume (*p* = 0.79; Figure 8B), cerebral edema (*p* = 0.41; Figure 8C) or midline shift (*p* = 0.39; Figure 8D) seen between sexes following stroke. SP levels in the serum were not significantly different between males and females (*p* = 0.41; Figure 8E). However, there was a significant increase in SP seen in the CSF (Figure 8F) of females when compared to males pre-stroke (*p* = 0.002), and at 1 (*p* < 0.0001), 3 (*p* < 0.0001) and 6 (*p* = 0.002) days post-stroke although there were no significant within group differences observed in the females at any time-point pre- or post-stroke (*p* = 0.18).

### Immunohistochemistry

#### Albumin

**FIGURE 9:**
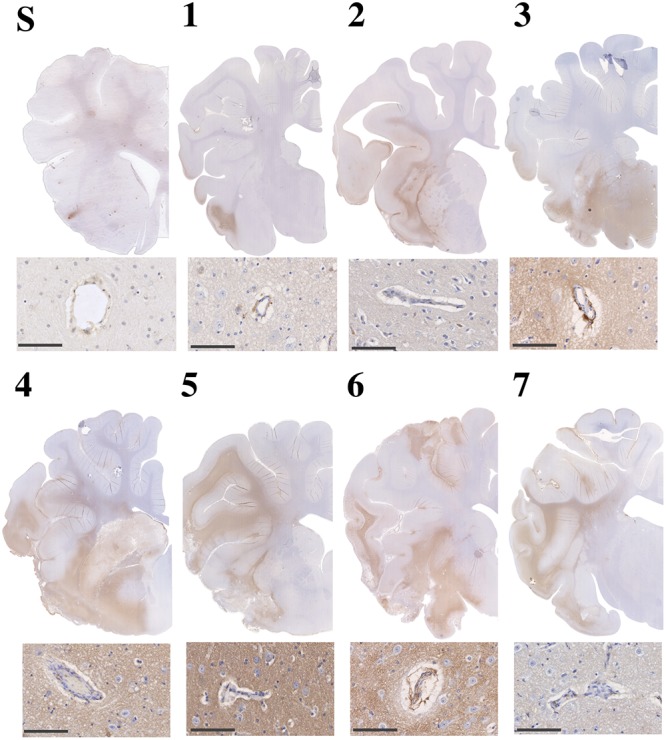
Albumin immunoreactivity in sham (S) and post-stroke animals at 1 (1), 2 (2), 3 (3), 4 (4), 5 (5), 6 (6) and 7 days (7) post-stoke. Enhanced albumin extravasation evolved over time following stroke and was most prominent at 4–6 days following tMCAO. This pattern of albumin staining was consistent with perivascular staining seen microscopically. Microscopic images scale bar 100 μm, 40× magnification.

There was minimal evidence of albumin extravasation in sham tissue (Figure 9). Following stroke, marked albumin extravasation, indicative of BBB breakdown and subsequent vasogenic cerebral edema formation, was observed both macroscopically within the infarcted territory and microscopically in the perivascular tissue of the infarct, most prominent of which was at 5 and 6 days post-stroke.

#### SP

**FIGURE 10:**
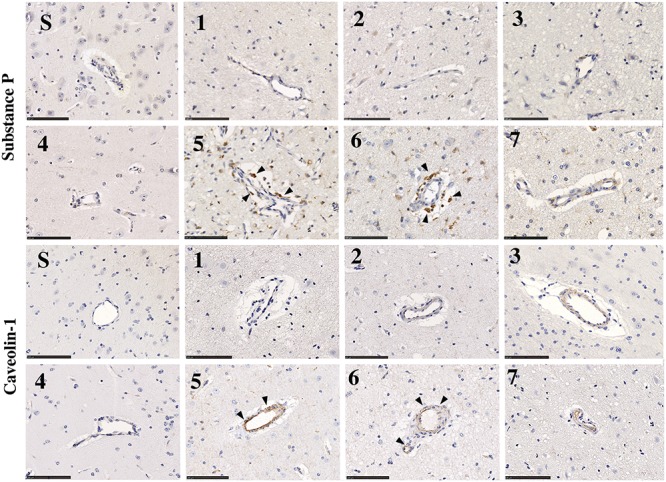
SP and caveolin-1 immunoreactivity in sham (S) and post-stroke animals at 1 (1), 2 (2), 3 (3), 4 (4), 5 (5), 6 (6), and 7 days (7) post-stoke. There was no identifiable immunoreactivity of SP or caveolin-1 in sham animals (S). Low levels of SP immunoreactivity were seen perivascularly at 1–4 days post-stroke. At 5 and 6 days following transient MCAO, there was an increase in SP seen in the perivascular tissue of the ischemic penumbra in the affected hemisphere. This increase in SP was concordant with an increase in caveolin-1 immunoreactivity perivascularly, which was most evident at 5–6 days. Arrows indicate increased immunoreactivity of SP and cav-1 seen surrounding vessels. Scale bar 100 μm, 40× magnification.

Little to no perivascular SP immunoreactivity was observed in sham tissue (Figure 10, Substance P, S). Following stroke, a marked increase in perivascular SP immunoreactivity was observed in tissue at 5 and 6 days, which was still evident at 7 days, although less pronounced.

#### Cav-1

Low levels of caveolin-1 immunoreactivity were observed in sham tissue (Figure 10, Caveolin-1, S). Following stroke, an increase in perivascular caveolin-1 immunoreactivity was identifiable which was particularly evident at 5 and 6 days post-stroke.

## Discussion

This study is the first to describe a surgical approach to ovine tMCAO with reperfusion. Through this study we have successfully developed a model that conserves tissue and permits post-operative recuperation, allowing for investigation into the pathophysiology of ischemic stroke, in particular post-stroke complications such as BBB disruption, development of cerebral edema and elevated ICP. This method of transient stroke induction provides a clinically relevant model in which promising novel treatments targeting stroke and its complications can be screened.

Although a number of studies have reported raised ICP following stroke in small animal models (Beard et al., 2015; Murtha et al., 2015) and large animal species at early time-points (Wells et al., 2015) this study is the first in which ICP was recorded out to 7 days post-stoke in a large gyrencephalic species. Using this model we have successfully characterized the temporal profile and established that ICP peaks at 5–6 days following ovine transient MCAO. These findings are comparable to the human patients, where ICP is shown to peak 3–5 days post-stroke, indicating that our model reliably replicates the clinical situation (Battey et al., 2014). The modest difference in the time course between the ovine model and humans may reflect the additional time taken to restore intracranial dynamics following surgery due to CSF aspiration required for optimal visualization of the proximal MCA. Indeed, a closed skull approach to MCAO where ICP dynamics are maintained through the induction of stroke may yield a timeline of ICP changes that directly align with clinical pathogenesis, however, this was not possible due to the complex ovine extracranial cerebrovasculature, in particular the rete mirabile, which precludes vascular access via the ICA for insertion of a balloon catheter, autologous clot or other occlusive agent.

Regardless, at 5–6 days post-stroke we consistently observed elevated ICP readings above 20 mmHg. In the clinic, ICP readings of ≥20 mmHg warrant investigation for possible treatment and intervention (Lavinio and Menon, 2011; Battey et al., 2014), as persistently elevated ICP is associated with poor outcome following ischemic stroke, widely accepted as being attributable to the development of edema in the cerebral parenchyma (Ayata and Ropper, 2002). Despite this, conflicting studies have reported an elevation of ICP in the absence of cerebral edema following small ischemic stroke in rats (Beard et al., 2015; Murtha et al., 2015). However, the underlying pathophysiology of increased ICP was not reported and requires further investigation. Furthermore, in the case of larger hemispheric strokes seen clinically, such as that of proximal MCAO or ICA occlusion, there is a positive correlation between cerebral edema and elevated ICP (Engelhorn et al., 2002). However, we did not observe such a relationship in this study, which may reflect the within group variability and different cohorts that were required to populate the time course. Furthermore, when measuring elevated ICP following stroke, the influence of autoregulation must also be taken into consideration. The ability to maintain cerebral homeostasis plays an important role in moderating the pathogenesis of elevated ICP. A loss of autoregulation has been shown to enhance the progression of ischemia and development of cerebral edema, and thus plays an important role during MCA reperfusion (Dohmen et al., 2007). It is proposed that autoregulation may be enhanced quite early following stroke, and become dampened at 6 days post-stroke (Reinhard et al., 2012), in keeping with our observations of significantly elevated ICP at 6 days post-stroke. Furthermore, elevated ICP is associated with a decreased ability to autoregulate following head injury, indicating a strong positive correlation between the two (Reinhard et al., 2012).

We observed an evolution of cerebral edema over time following stroke, although changes were not significant between time-points. Nevertheless, it is widely accepted that the increased volume of the brain as a result of cerebral edema positively correlates with subsequent elevations in ICP (Battey et al., 2014; Beard et al., 2015; Brogan and Manno, 2015). The development of vasogenic cerebral edema in particular occurs following loss of integrity of the BBB, permitting the abnormal extravasation of proteins across the damaged barrier. This creates an osmotic drive pulling water from the vasculature into the extracellular cerebral parenchyma, leading to an overall increase in brain volume and concomitant rise in ICP (Battey et al., 2014). Our study was able to confirm that there was disruption to BBB via visualization of the GAD contrast medium within the brain parenchyma, suggesting that the cerebral edema seen was vasogenic in nature. This finding was substantiated by the increased extravasation of albumin following tMCAO, which was most prominent at 5–6 days post-stroke. Albumin is unable to cross the intact barrier under physiological conditions due to its large size and structure (Quinlan et al., 2005). Previous studies have established that albumin crosses the barrier via the transcellular pathway, facilitated by caveolae (Abbott et al., 2006), of which caveolin-1 (cav-1) is an integral component (Nag et al., 2007). Indeed, increased barrier permeability in association with caveolin-1 upregulation was observed in the absence of tight junction breakdown, confirming the integral role of caveolae in the maintenance of barrier integrity (Povlishock et al., 1978; Nag et al., 2007). Our IHC findings of increased cav-1 immunoreactivity at 5–6 days post-stroke corroborate this, providing a potential mechanism driving sustained barrier permeability and the evolution of cerebral edema. Furthermore, the volumetric enlargement of the brain as a result of vasogenic edema leads to displacement of cerebral tissue and subsequent shift of the midline, a critical state in which compression of adjacent brain structures precedes potentially fatal tonsillar herniation (Huttner and Schwab, 2009). We observed significant midline shift at 5 days following stroke in keeping with elevated ICP. As such, our findings of enhanced GAD, albumin and cav-1 immunoreactivity at 5–6 days post-stroke suggest a loss of barrier integrity at this time-point, precipitating the development of vasogenic cerebral edema, extensive midline shift and the subsequent rise in ICP seen in this model.

We also demonstrated that serum SP levels were increased at 7 days following stroke when compared to pre-stroke values. This followed a non-significant decline in serum SP at 4 h which continued to decline out to 16 h following injury. This suggests a peak increase in the levels of serum SP may occur ahead of the 4 h-post-stroke sampling time-point. Indeed, rodent TBI studies have reported an early peak in plasma SP levels at 30 min post-injury but a decline by 5 h post-injury (Donkin and Vink, 2010) Similarly, SP levels were increased in serum samples obtained within 12 h following the onset of both clinical stroke (Bruno et al., 2003; Lorente et al., 2016) and TBI (Lorente et al., 2015), with higher levels associated with early mortality. Although some discrepancy between experimental and clinical time-points exists, it is important to acknowledge that it is difficult to draw firm comparisons given the complications in obtaining clinical samples at acute time-points. We also examined SP levels in the CSF following stroke, though observed no differences at 1, 3, or 6 day time-points. Although females consistently had higher CSF SP levels compared to males at all post-stroke time-points, there was no significant change in these levels post-stroke. This suggests that females have higher baseline levels of SP. Indeed, gender differences in the SP response have previously been observed (Pittenger et al., 2016; Simões et al., 2018). A comprehensive sampling regime, including sampling at 4 and 6 days post-stroke for serum and 4 and 5 days post-stroke for CSF, would be required to populate a detailed time-course of changes in SP in the serum and CSF in the setting of ischemic stroke, and may reveal changes beyond those observed in the current cohort. Nevertheless, the increase in serum SP levels we observed, in conjunction with the increased barrier permeability and vasogenic edema seen with albumin IHC and MRI, support a potential role for SP in the genesis of cerebral edema and development of elevated ICP in this model. Indeed, our rodent stroke studies support this (Turner et al., 2006, 2011; Corrigan et al., 2012, 2016; Turner and Vink, 2012, 2014) and corroborates our recent work demonstrating the efficacy of NK1-r antagonist treatment in reducing ICP in an ovine model of permanent MCAO (Sorby-Adams et al., 2019). Taken together, these findings suggest that neurogenic inflammation occurs following ischemic stroke and may contribute to the increased BBB permeability and elevated ICP observed, suggesting that blocking the action of SP with a tachykinin NK1-r antagonist may be an effective strategy to mitigate these post-stroke complications and thus improve outcome.

## Limitations and Future Directions

Although the findings of this study demonstrate successful model development and clearly show the evolution of ICP following stroke, limitations must be acknowledged. It must firstly be recognized that human patients are rarely under anesthesia when suffering a stroke. This is of particular importance due to the implied effects of anesthesia, both protective and deleterious. Ketamine has been shown to have neuroprotective effects (Hudetz and Pagel, 2010), whilst isoflurane is associated with respiratory depression, decreased MABP and thus diminished oxygen supply to neuronal cells (Murr et al., 1993), exacerbating cerebral ischemia. These limitations have been discussed in detail in our previous studies (Wells et al., 2012, 2015), however, due to the nature of the surgical procedure, anesthesia is unavoidable (Lin et al., 1997). The decision to maintain animals on a combination of ketamine and isoflurane was made on the basis of prolonged duration (4–5 h) of surgical procedures. The anesthetic combination produces a countering effect, eliminating the neuroprotective effect of pure ketamine and the respiratory distress associated with pure isoflurane, thus reducing confounding factors of anesthetic agents administered alone (Schifilliti et al., 2010).

There was a large within group variability, particularly for the MRI parameters, seen at each of the time-points following stroke. This may reflect differences in collateral flow or autoregulation capacity within the individual animals. Indeed, there is a considerable degree of variation in the severity and timing of cerebral edema in human patients following stroke. Furthermore, there may be some variability due to the severity of stroke in addition to the relative age of the sheep compared with the greater stroke population. In this study we used sheep aged 18–24 months, which are the equivalent to mid-adulthood in humans, whereas clinical stroke most commonly occurs in those over 60 years of age. Furthermore, the exclusion of experimental animals due to anesthetic complications or lack of reperfusion decreased the total sample size. This could be addressed by increasing the overall sample size for each group to increase statistical power. It must also be acknowledged that we used different cohorts of animals to generate the stroke ICP data, sham ICP data and serum/CSF for the ELISAs. This led to an inability to run true correlations between groups, for example between the SP and ICP datasets, limiting the conclusions we can draw regarding the potential relationship between serum/CSF SP and ICP. Furthermore, there was some discrepancy seen between elevated ICP and edema at 5–6 days in the first cohort and elevated serum SP seen at 7 days in the second cohort. Our findings suggest that SP is involved in the evolution of edema, however, given that different cohorts used in this study it was difficult to establish a definitive relationship between the two. As such, the differences in the time course of SP elevations and increased ICP are most likely attributable to the different cohorts used for each of these outcome measures.

In order to reduce the number of animals required we utilized a cohort of sham animals that underwent sham surgery immediately followed by ICP monitoring. We acknowledge that we would have preferentially used the same regime as the stroke cohort but it was not possible to generate an additional sham control cohort for this study alone. When measuring ICP, values would ideally be obtained from the same animal from 1–7 days post-stroke to look at the evolution of pressure individual to that animal. This could be addressed through the use of a telemetric system to remotely and non-invasively measure ICP. As sheep are quadrupeds however, some question remains regarding the impact of head position, in particular when feeding, on ICP measurements obtained using this method. Furthermore, the use of individual cohorts of animals benefited this study by allowing us to harvest the brain at specific time-points following stroke and perform IHC accordingly, an important aspect to understanding the evolution of the injury. The reliable measurement of cerebral edema on MRI at a single time-point is somewhat contentious, as the hyperintensity of vasogenic edema is difficult to distinguish from the lesion itself. The measurement of the entire FLAIR lesion volume therefore includes areas of infarction as well as edema and is therefore an indirect measure of edema volume. A more robust approach to measuring edema would be to undergo sequential MRI’s and evaluate the change in diffusion lesion volume between 2 time points following reperfusion, which was not feasible in this study due to cost. Furthermore, although GAD was utilized, T1 weighted MRI images were only taken at a single time-point following administration. As such, it is difficult to obtain a quantitative measure of GAD extravasation across the barrier. A more robust approach to this would be to obtain dynamic contrast enhanced (DCE) and dynamic susceptibility contrast (DSC) sequences to generate clinically relevant measurements of barrier permeability. Finally, although gender differences were examined in this study the hormonal status of the female animals at the time of stroke and monitoring period was not known.

## Conclusion

In this study, we have successfully developed a surgical approach to tMCAO with reperfusion in the sheep that permits recovery and the examination of long-term time-points following stroke. In this novel model we have determined the temporal profile of ICP, showing that ICP peaks at 5–6 days following stroke, in conjunction with midline shift, an evolution of cerebral edema, increased serum SP levels, enhanced SP immunoreactivity and increased albumin extravasation. These results suggest a role of SP in BBB disruption and the subsequent genesis of cerebral edema and associated rise in ICP following stroke. As such, agents targeting elevated ICP and cerebral edema would be most effective when administered prior to 6 days post-stroke, or as early as possible following stroke-onset. We conclude that this ovine model provides a promising platform to screen novel treatments targeting post-stroke complications and verify treatment efficacy prior to clinical assessment.

## Data Availability

All datasets generated for this study are included in the manuscript and/or the supplementary files.

## Ethics Statement

All experimental procedures were approved by the Animal Ethics Committees of The University of Adelaide (M-2014-015) and South Australian Health and Medical Research Institute (SAHMRI SAM 3; SAM 141). Experiments were conducted in accordance with the Australian National Health and Medical Research Council code of practice for the care and use of animals for scientific purposes (8th edition, 2013).

## Author Contributions

AS-A, RV, RT, and AL conceived and designed the experiments. AS-A, RT, ET, OM, and LE performed the experiments. AS-A, LE, JH, and NY analyzed the data. AS-A and RT wrote the manuscript.

## Conflict of Interest Statement

The authors declare that the research was conducted in the absence of any commercial or financial relationships that could be construed as a potential conflict of interest.
